# Eight Weddings and Six Funerals: An fMRI Study on Autobiographical Memories

**DOI:** 10.3389/fnbeh.2018.00212

**Published:** 2018-09-18

**Authors:** Francesca Benuzzi, Daniela Ballotta, Giacomo Handjaras, Andrea Leo, Paolo Papale, Michaela Zucchelli, Maria Angela Molinari, Fausta Lui, Luca Cecchetti, Emiliano Ricciardi, Giuseppe Sartori, Pietro Pietrini, Paolo Frigio Nichelli

**Affiliations:** ^1^Department of Biomedical, Metabolic and Neural Sciences, University of Modena and Reggio Emilia, Modena, Italy; ^2^Center for Neurosciences and Neurotechnology, University of Modena and Reggio Emilia, Modena, Italy; ^3^Molecular Mind Lab, IMT School for Advanced Studies Lucca, Lucca, Italy; ^4^Department of Psychology, University of Bologna, Bologna, Italy; ^5^Azienda Unità Sanitaria Locale di Modena, Modena, Italy; ^6^Department of Psychology, University of Padua, Padua, Italy

**Keywords:** autobiographical memory, individual differences, emotional valence, retrievial, multivariate analysis

## Abstract

“Autobiographical memory” (AM) refers to remote memories from one's own life. Previous neuroimaging studies have highlighted that voluntary retrieval processes from AM involve different forms of memory and cognitive functions. Thus, a complex and widespread brain functional network has been found to support AM. The present functional magnetic resonance imaging (fMRI) study used a multivariate approach to determine whether neural activity within the AM circuit would recognize memories of real autobiographical events, and to evaluate individual differences in the recruitment of this network. Fourteen right-handed females took part in the study. During scanning, subjects were presented with sentences representing a detail of a highly emotional real event (positive or negative) and were asked to indicate whether the sentence described something that had or had not really happened to them. Group analysis showed a set of cortical areas able to discriminate the truthfulness of the recalled events: medial prefrontal cortex, posterior cingulate/retrosplenial cortex, precuneus, bilateral angular, superior frontal gyri, and early visual cortical areas. Single-subject results showed that the decoding occurred at different time points. No differences were found between recalling a positive or a negative event. Our results show that the entire AM network is engaged in monitoring the veracity of AMs. This process is not affected by the emotional valence of the experience but rather by individual differences in cognitive strategies used to retrieve AMs.

## Introduction

The expression Autobiographical memory (AM) refers to remote memories from one's own life which are characterized by a sense of subjective time, autonoetic awareness (Tulving, 2002), and feelings of emotional re-experience (Tulving, 1983; Tulving and Markowitsch, 1998). AM is part of episodic memory (i.e., the conscious recollection of experienced events), as opposed to semantic memory-i.e., the conscious recollection of factual information and general knowledge about the world (Tulving, 2002). Neuropsychological and neuroimaging data support this notion of multiple systems of memory, each specialized in processing distinct types of information (Vargha-Khadem et al., 1997; Cipolotti and Maguire, 2003) and subserved by distinct, functionally independent neural networks (Gabrieli, 1998; Cabeza and Nyberg, 2000; Tulving, 2002).

As a matter of fact, neuropsychological studies support the functional dissociation between these memories: patients with medial temporal lobe lesions are defective in AM recall, but not in semantic memory tasks (Vargha-Khadem et al., 1997; Tulving and Markowitsch, 1998; Gadian et al., 2000). Conversely, patients with semantic dementia, who show damage in fronto-temporal regions, are impaired in semantic memory tasks (Neary et al., 1999), whereas their AM is relatively spared (Snowden et al., 1994; McKinnon et al., 2006).

More recently, neuroimaging studies have disentangled the functional characteristics of the neural networks mediating specific memory systems. The left inferior prefrontal cortex and left posterior temporal areas are in general recruited during semantic retrieval (Vandenberghe et al., 1996; Wiggs et al., 1999; Graham et al., 2003), whereas right dorsolateral prefrontal areas subserve episodic retrieval (Cabeza et al., 2004; Düzel et al., 2004; Gilboa, 2004). With respect to AM, functional neuroimaging studies focused on voluntary retrieval processes that involve different forms of memory and cognitive functions. In particular, recovering an autobiographical event requires a prolonged and effortful memory search about one's own life, combined with the retrieval of specific episodic knowledge about its contextual information. The retrieved memory content typically includes emotions and visual images, and is mediated by inferential and monitoring cognitive processes (Cabeza and St Jacques, 2007).

A meta-analysis paper showed that, because of the multi-modal nature of AM retrieval and of the heterogeneity of the tasks used in literature, different regions emerge during recollection (Svoboda et al., 2006). However, a core neural network for AMs comprises the left lateral prefrontal cortex (l-PFC) for search and controlled processes; the medial prefrontal cortex (m-PFC) for self-referential processes; the hippocampus and the retrosplenial cortex for recollection; the amygdala for emotional processing; the occipital and cuneus/precuneus regions for visual imagery, and the ventromedial PFC (vm-PFC) regions for feeling-of-rightness and monitoring (Cabeza and St Jacques, 2007).

Two additional issues are relevant for AM. First, AMs often exhibit a richer emotional content as compared to episodic and semantic memories. In particular, emotional life events are recalled better than non-emotional events (Holland and Kensinger, 2010). Second, several neuroimaging studies demonstrated a significant individual variability in AMs performance (Rypma et al., 2002; Schaefer et al., 2006; Miller and Van Horn, 2007). Typically, most of these studies evaluated the modulation of brain areas commonly activated across subjects, and only a few studies considered the individual variability across the whole brain (McGonigle et al., 2000; Feredoes and Postle, 2007; Seghier et al., 2008).

In spite of the importance of the mechanisms underlying the successful recollection from AM, only a few studies previously investigated this issue (Gilboa et al., 2004; Greenberg et al., 2005; Cabeza and St Jacques, 2007; Chen et al., 2017). Rather, many authors questioned whether brain functional patterns could differentiate between true memory, false memory (a common type of memory distortion in which individuals incorrectly believe they have already encountered a novel object or event), and deception. Regions within the prefrontal cortex have been related to these memory monitoring activities (Cabeza and St Jacques, 2007). Nonetheless, to the best of our knowledge, only one study evaluated recognition from AM (Harris et al., 2008). However, the authors used a wide range of stimuli (autobiographical, mathematical, geographical, religious, ethical, semantic, and factual) and results were presented irrespectively of the kind of memory involved.

The present single-event fMRI study was designed to determine whether neural activity within the AM network, as identified by previous neuropsychological and neuroimaging studies, would recognize memories of real autobiographical events. Moreover, we examined whether retrieval of positive and negative emotional events from AM would exert distinctive effects on brain response. Specifically, we asked subjects to recall a highly emotional personal event (either her wedding or the funeral of a close relative) in a pre-scan semi-structured interview. During scanning, subjects were presented with sentences referring to a detail of the event recalled and were asked to indicate whether the detail actually belonged (true) or not (false) to their AMs. Using a multivariate technique (Mitchell et al., 2008), we aimed at evaluating the neural network in each individual subject independently, so that we could identify both the time points at which the successful recollection occurred and the network involved in the process. Then, results from each subject were combined to identify the brain regions involved in the common cognitive mechanism underlying AM, thus accounting for individual differences in the recollection processes.

## Materials and methods

### Subjects

Inclusion criteria were: right-handed healthy females with no history of neurological or psychiatric diseases; no subject took any psychiatric medication at the time of the study; age 30–45 years; having experienced either a highly positive (own wedding, being still married at the time of the experiment) or a highly negative (funeral of a loved one, who died suddenly) event in the recent past (range: 2–8 years). Consequently, 14 subjects (mean age 37 ± 7 years; mean school-age 17 ± 2) were enrolled. This final group included: personnel from the University of Modena and Reggio Emilia staff, acquaintances and relatives of the authors. Only female volunteers participated to the study, as data in the literature indicate that gender influences memory, and particularly the emotional modulatory mechanism on memory storage (Cahill, 2010). All participants gave their written informed consent after the study procedures and potential risks had been explained. The study was conducted under protocols approved by the Local Modena Ethical Committee, in accordance with the ethical standards of the 2013 Declaration of Helsinki.

#### Pre-scan interview session

From 2 to 8 days before fMRI scanning, a detailed description of highly emotional events was collected using a custom-made semi-structured interview. Indeed, the “pre-scan interview method” could be particularly useful to evaluate the common and individual neural network for retrieving AMs in neuroimaging studies. Eight participants were asked to describe a positive event (i.e., their wedding), whereas six participants to recall a negative event (i.e., the funeral of a loved one). The interview about the wedding day consisted of 54 questions, organized in 4 different categories concerning: 1. the ceremony; 2. the wedding dress; 3. the wedding party; 4. the honeymoon. Four categories were also included in the funeral day's interview (32 questions): 1. the deceased's physical description at the time of his/her death; 2. the announcement of the death; 3. the last meeting; 4. the funeral. The answers were used to compose a true story. A second false story was written, modifying some details of the true story (e.g.,: “We got married *in April*”: true; “We got married *in September*”: false). The true stories consisted of information stored in the autobiographical memory (AM) of the participants, whereas the details of the false stories did not belong to their AM.

### Image acquisition and experimental setup of the fMRI session

Brain activity was measured using fMRI with a three-run event-related design (gradient echo echoplanar images, Philips Achieva 3T, TR 2.0 s, FA: 80°, TE 35 ms, 30 axial slices, 80 × 80 acquisition matrix, 3 × 3 × 4 mm voxel). High-resolution T1-weighted spoiled gradient recall (*TR* = 9.9 ms, *TE* = 4.6 ms, 170 sagittal slices, 1 mm isovoxel) images were obtained for each participant to provide detailed brain anatomy.

Behavioral responses were collected during the scanning sessions by means of a custom-made software developed in Visual Basic 6 (http://digilander.libero.it/marco_serafini/stimoli_video/). The same software was used to present stimuli via IFIS-SA System (MRI Device Corporation, WI, USA) remote display.

**Figure 1:**
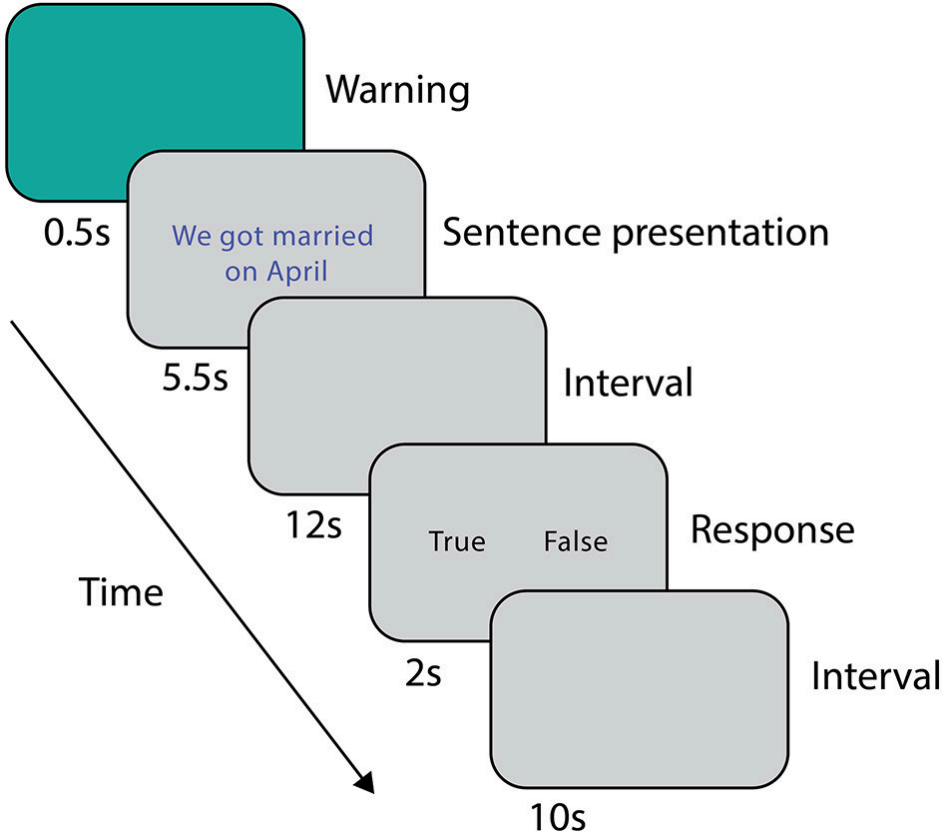
Experimental protocol for the fMRI scan session.

During the scanning session, prior to the fMRI acquisition, subjects were asked to read both stories (i.e., the true and the false one) twice, in order to avoid the novelty effect of the incorrect information (Schomaker and Meeter, 2015). The order of presentation of the stories was counterbalanced between subjects. The experimental stimuli were sentences representing a true or a false detail of the event described in the stories. The false and true item referring to the same AM detail differed only in one feature (i.e., *He died in May* vs. *He died in April*; *My wedding dress was white* vs. *My wedding dress was ivory*). During scanning, after a warning cue lasting 0.5 s, subjects were presented with a sentence (5.5 s). After a 12 s interval, subjects were asked to indicate whether the sentence belonged (true, T) or not (false, F) to their autobiographical memory by pressing one of two buttons on the keypad (2 s, Figure 1), followed by 10 s of inter-trial interval. Response times and accuracies were recorded. A total of 48 sentences (24 T and 24 F) were randomly presented to each subject in three runs. At the beginning and at the end of each run, a fixation cross was presented for 30 s to obtain a baseline measure of brain activity. Overall, each run lasted about 9 min. The true-false responses given during scanning were subsequently used for the behavioral and functional analyses.

#### Behavioral analysis

A two-way ANOVA was performed on the response times with the following factors: *group* (two levels, wedding and funeral) and *response* (two levels, true and false). Significance threshold was set at p < 0.05. Analyses were performed using SPSS 18 (SPSS Inc.).

#### fMRI data preprocessing

The AFNI software package was used to analyze functional imaging data (Cox, 1996). All volumes from the different runs were processed to remove spikes (*3dDespike*), temporally aligned (*3dTshift*), corrected for head movements (*3dvolreg*), spatially smoothed (*3dmerge*, Gaussian kernel 5 mm, FWHM) and normalized. Motion spikes were estimated through the evaluation of Framewise Displacement (FD) implemented in FSL (Jenkinson et al., 2012), with a cutoff of 0.6 mm (Power et al., 2012). Subsequently, a generalized least squares regression was performed (*3dREMLfit*) to model the motion spikes, movement parameters, signal trends and the temporal correlation structure with an ARMA(1,1) model, thus to remove nuisance signals from the data. Then, the residual signal for each voxel was normalized by subtracting the mean and dividing the result by its standard deviation. Afterwards, for each trial, the signal time points from the onset of the sentence to the motor response, were extracted and included in the multivariate analysis. To improve signal-to-noise ratio, a central moving average was computed (“temporal smoothing”) (Friston et al., 1995; Strappini et al., 2017) by averaging the value of each point in time (“reference point”) and the value of the two points on either side of the reference point. By this procedure, we generated seven overlapping windows, from 2 to 14 s after sentence onset. The duration of the explored window was decided following previous studies which showed that the retrieval of detailed autobiographical memories can spread over a long time (e.g., up to 20 s) (Svoboda et al., 2006), but also in order to avoid any overlap with the motor response.

Subsequently, single subject time series data were registered to the MNI152 standard space using the nonlinear registration implemented in AFNI (*3dQWarp*), and the acquisition matrix was resampled to a 3 mm iso-voxel. Finally, to reduce computational effort in the subsequent steps, a spatial mask was applied to select gray matter voxels only.

#### Single-subject decoding analysis

Since we were interested in selecting the best subset of voxels with the highest discrimination ability in distinguishing between “true” and “false” responses, we used a modified version of the procedure originally adopted by Mitchell et al. (2008) and already validated on different datasets (Handjaras et al., 2016; Leo et al., 2016). Briefly, a machine-learning algorithm predicted the fMRI activation in the brain as a weighted sum of images, each one generated from a behavioral matrix (here, a binary vector which defined the “true” and “false” responses). In detail, a regression analysis, performed within a leave-two-stimuli-out cross-validation procedure, produced a learned scalar parameter that specifies the degree to which the dimension related to the truthfulness of the memories modulates the voxels activity. Hence, for each iteration of the cross-validation procedure, the model was first trained with 46 out of 48 stimuli (i.e., 23 “true” and 23 “false”), then only the 2,000 voxels that showed the highest coefficient of determination R^2^ and with a cluster size larger than 20 voxels (to remove small isolated clusters) were considered. Once trained, the resulting algorithm was used to predict the fMRI activation within the selected 2,000 voxels of the two left-out stimuli (one related to a “true,” one to a “false” response). Afterward, prediction accuracy was evaluated with a simple match between the predicted and the real fMRI activations of the two left-out stimuli using cosine similarity. This leave-two-out procedure was iterated 576 times, training and testing all possible stimulus pairs between the true and false items. A bootstrapping procedure was used to measure the standard error of the accuracy (1,000 iterations) (Efron and Tibshirani, 1993). The algorithm for the single-subject decoding analysis was applied for each subject and time point (i.e., from 2 to 14 s after sentence onset), thus generating an accuracy value and a decoding map with the subset of brain voxels used during the procedure.

**Figure 2:**
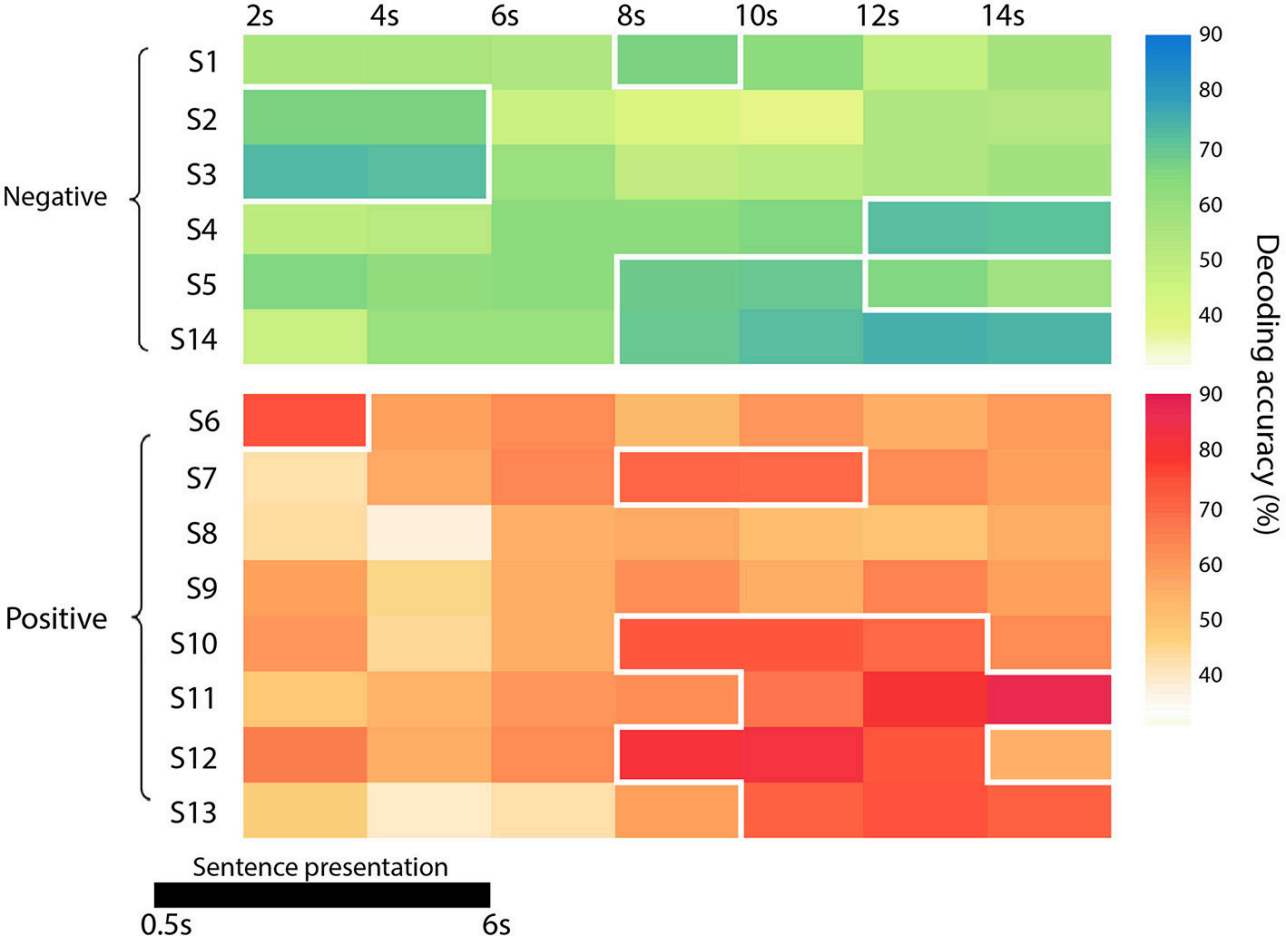
Diagram representing the accuracy of each subject and group (in green the negative one -the funeral of a loved one- and in red the positive event -wedding), at each time point. Significant time points (*p* < 0.05) are marked with a white border.

The single-subject accuracy was tested for significance against the null distribution of accuracies generated with a permutation test based on the same procedure defined above (Schreiber and Krekelberg, 2013; Handjaras et al., 2015). As the processing of false sentences does require the retrieval of information related to the true event counterpart, we adopted permutation tests: these are the most robust methods to assess statistical significance in conditions, such as our experiment, where the chance level is not necessarily centered on 50% and where the degrees of freedom are unknown, ranging between the number of the stimuli (i.e., 48) and the total number of comparisons (i.e., 576). Moreover, as the null distribution was always created upon individual brain activity in each subject, the significance threshold reflected any possible bias in the data. Briefly, in each subject and time point, a null distribution of accuracies was built by shuffling the behavioral matrix during the training phase. The procedure was repeated 100 times (Winkler et al., 2016) for each time point, leading to a null distribution of 700 accuracy values across the whole time window. Each single-subject accuracy was therefore tested against the null distribution of accuracy values to identify a common significance threshold across the time window (one-sided rank test, *p* < 0.05; Table 1 and Figure 2).

**Table 1:** Table representing the raw accuracy value, its standard error and *p*-value of each subject and group at each time point.

**Group**	**Subject**	**Time window**						
		**2 s**	**4 s**	**6 s**	**8 s**	**10 s**	**12 s**	**14 s**
Funerals	*sub1*	54.3%, *p* = n.s.	54.7%, *p* = n.s.	53.3%, *p* = n.s.	**66.2** ± **1.7%**, ***p*** = **0.041**	62.2%, *p* = n.s.	47.0%, *p* = n.s.	55.7%, *p* = n.s.
	*sub2*	**65.7** ± **1.8%**, ***p*** = **0.044**	**65.7** ± **1.8%**, ***p*** = **0.044**	45.4%, *p* = n.s.	39.7%, *p* = n.s.	36.8%, *p* = n.s.	53.0%, *p* = n.s.	52.2%, *p* = n.s.
	*sub3*	**73.0** ± **1.7%**, ***p** =* **0.009**	**72.0** ± **1.8%**, ***p** =* **0.011**	60.0%, *p* = n.s.	48.2%, *p* = n.s.	50.3%, *p* = n.s.	53.0%, *p* = n.s.	56.4%, *p* = n.s.
	*sub4*	49.1%, *p* = n.s.	51.0%, *p* = n.s.	62.6%, *p* = n.s.	62.2%, *p* = n.s.	65.4%, *p* = n.s.	**72.0** ± **1.7%**, ***p** =* **0.013**	**71.1** ± **1.7%**, ***p** =* **0.019**
	*sub5*	64.9%, *p* = n.s.	61.7%, *p* = n.s.	62.7%, *p* = n.s.	**68.3** ± **1.9%**, ***p** =* **0.040**	**68.9** ± **1.7%**, ***p** =* **0.030**	65.3%, *p* = n.s.	57.6%, *p* = n.s.
	*sub14*	46.8%, *p* = n.s.	59.9%, *p* = n.s.	59.8%, *p* = n.s.	**69.0** ± **1.8%**, ***p** =* **0.021**	**71.9** ± **1.7%**, ***p** =* **0.009**	**74.1** ± **1.8%**, ***p** =* **0.006**	**73.2** ± **1.8%**, ***p** =* **0.007**
Weddings	*sub6*	**74.6** ± **1.5%**, ***p** =* **0.017**	57.4%, *p* = n.s.	62.2%, *p* = n.s.	52.0%, *p* = n.s.	59.3%, *p* = n.s.	55.3%, *p* = n.s.	58.8%, *p* = n.s.
	*sub7*	42.0%, *p* = n.s.	56.0%, *p* = n.s.	63.0%, *p* = n.s.	69.7 ± 1.4%, *p* = 0.014	68.9 ± 1.6%, *p* = 0.021	62.3%, *p* = n.s.	57.3%, *p* = n.s.
	*sub8*	43.0%, *p* = n.s.	37.0%, *p* = n.s.	53.8%, *p* = n.s.	56.2%, *p* = n.s.	50.3%, *p* = n.s.	49.4%, *p* = n.s.	54.5%, *p* = n.s.
	*sub9*	57.2%, *p* = n.s.	44.5%, *p* = n.s.	55.0%, *p* = n.s.	61.6%, *p* = n.s.	55.2%, *p* = n.s.	64.3%, *p* = n.s.	57.3%, *p* = n.s.
	*sub10*	59.2%, *p* = n.s.	43.2%, *p* = n.s.	54.5%, *p* = n.s.	**72.6** ± **1.5%**, ***p** =* **0.016**	**72.6** ± **1.7%**, ***p** =* **0.016**	**68.8** ± **1.6%**, ***p** =* **0.033**	62.4%, *p* = n.s.
	*sub11*	48.4%, *p* = n.s.	53.2%, *p* = n.s.	59.9%, *p* = n.s.	61.0%, *p* = n.s.	**67.4** ± **1.9%**, ***p** =* **0.049**	**78.9** ± **1.6%**, ***p** =* **0.002**	**86.8** ± **1.4%**, ***p***<**0.001**
	*sub12*	64.8%, *p* = n.s.	55.3%, *p* = n.s.	61.9%, *p* = n.s.	**79.7** ± **2.3%**, ***p** =* **0.004**	**81.8** ± **2.2%**, ***p** =* **0.004**	**73.8** ± **2.5%**, ***p** =* **0.020**	53.5%, *p* = n.s.
	*sub13*	45.9%, *p* = n.s.	39.3%, *p* = n.s.	41.9%, *p* = n.s.	57.2%, *p* = n.s.	**71.0** ± **1.8%**, ***p** =* **0.034**	**74.4** ± **1.8%**, ***p** =* **0.011**	**70.9** ± **1.8%**, ***p** =* **0.034**

*Significant time points (p < 0.05) are marked in bold*.

#### Group level map

**Figure 3:**
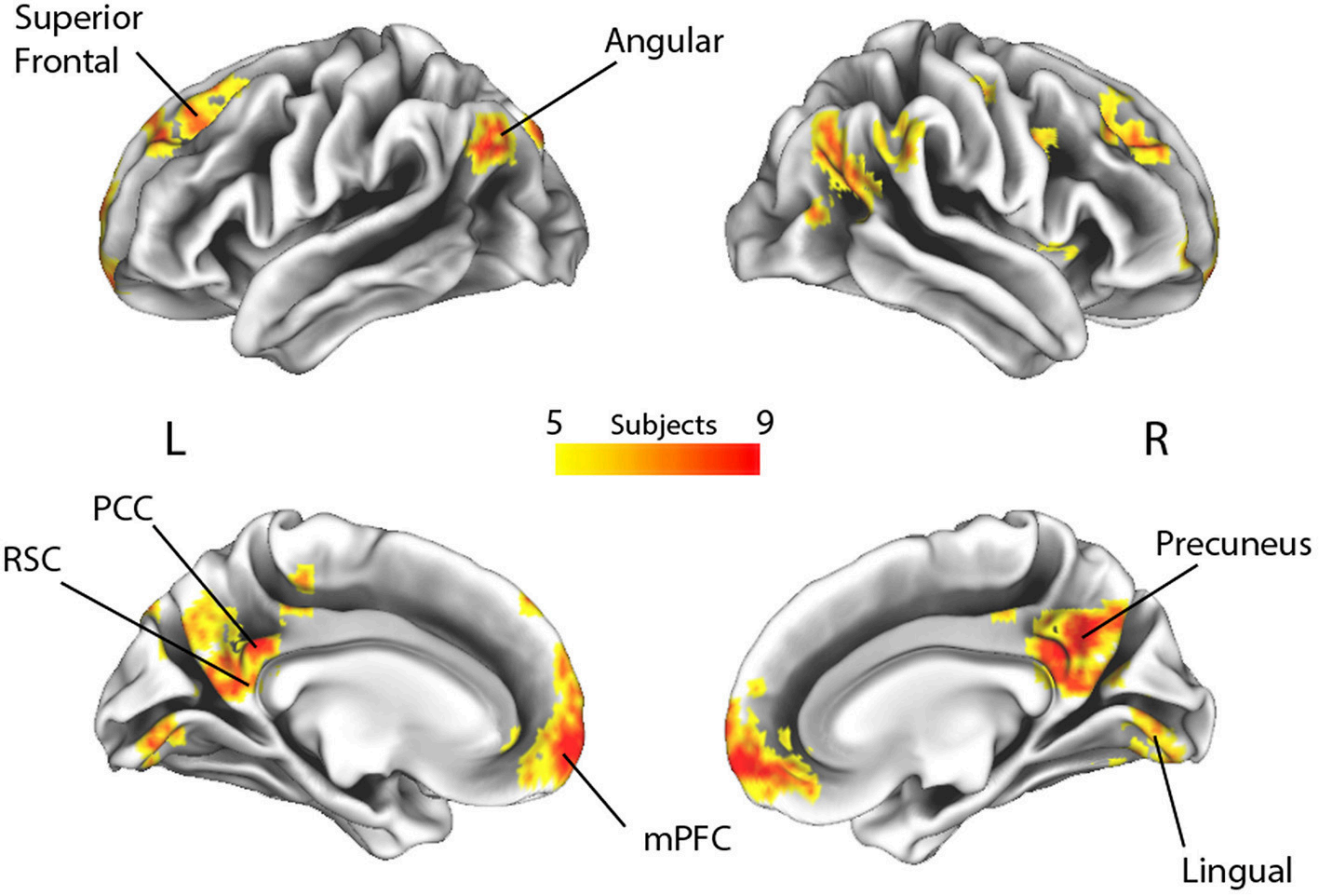
Spatial overlap of the decoding maps of all subjects across all time points (*p* > 0.33, which represents the probability of a voxel to be informative in at least 5 out of 14 subjects, irrespective of timing). L, Left; R, Right; RSC, retrosplenial cortex; PCC, posterior cingulate cortex; mPFC, medial prefrontal cortex.

Subsequently, to measure the spatial consistency of the regions involved in autobiographical memory processing, a posterior probability map was built across the time windows by combining the single subject decoding maps at the time point with the highest accuracy value. This procedure therefore merged the most informative voxels involved in the “true” and “false” responses irrespectively of the time at which the voxels were maximally engaged. We arbitrarily selected a threshold (*p* > 0.33, minimum cluster size of 20 voxels) that represented the probability of a voxel to be informative in at least 5 subjects out of 14 (Figure 3; Leo et al., 2016).

#### Assessing the reliability of the group level map

**Figure 4:**
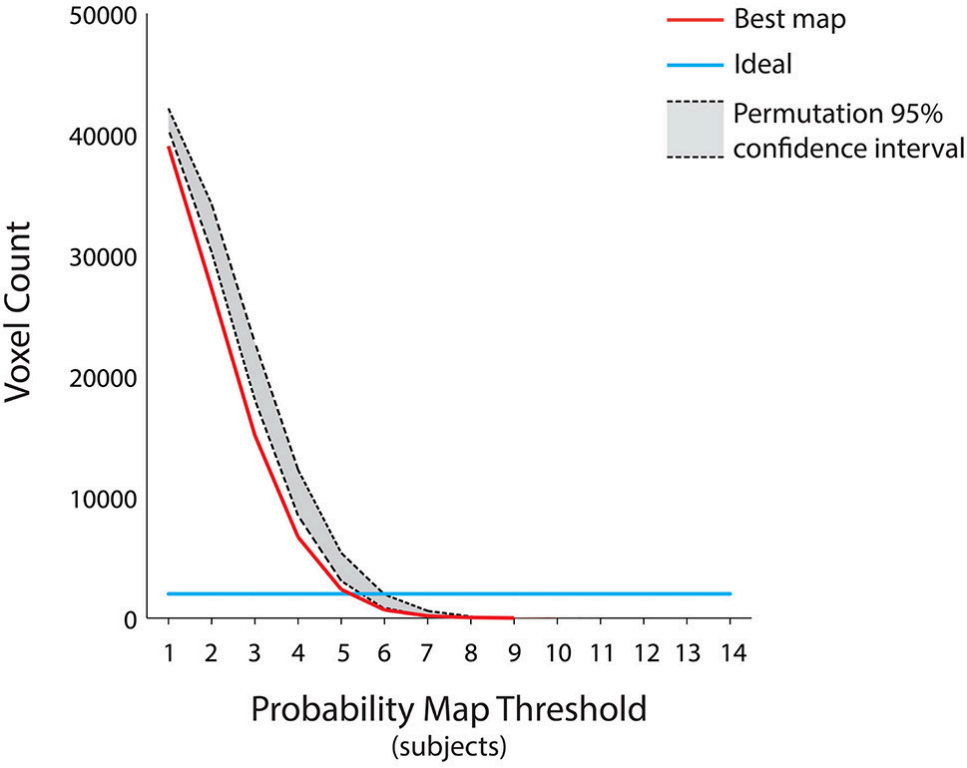
Assessment for the group level map. Since the group level map of Figure 3 was the result of the aggregation of the individual subject decoding maps at different time points, we further tested its sparseness using a permutation test by randomly combining the decoding maps at different time points across subjects and subsequently measuring the total number of voxels at each probability threshold (*p* < 0.05). The ideal group map (e.g., no variability across subjects) is represented by the light blue line, the group level map is represented by the red curve, whereas the 95% confidence interval of the null distribution is outlined in gray. The group level map has a number of voxels lower than the null distribution, irrespective of the chosen probability threshold. Moreover, all the group maps obtained by aggregating the subjects' decoding maps at each of the seven fixed time points fell within the null distribution area (*p* < 0.05).

This group level map was the result of the aggregation of the single subject most discriminative voxels at different time points, in order to account for the possibility that individual subjects processed autobiographical memory content with different retrieval times. Therefore, we further tested the sparseness of the map obtained from this procedure, as we reasoned that the cognitive mechanisms underlying the discrimination of “true” and “false” responses would engage the same brain regions across subjects. Theoretically (e.g., assuming no variability across subjects), the ideal group map should include the same 2,000 voxels of the decoding procedure across all subjects and probability thresholds, albeit at different time points (Figure 4). On these assumptions, a permutation test was built by randomly combining the decoding maps at different time points across subjects and subsequently measuring the total number of voxels at each probability threshold (10,000 iterations, *p* < 0.05) (Figure 4). We hypothesized that our group map should have the lower number of voxels, as compared to the null distribution, thus indicating that brain regions involved in the process remained significantly stable across subjects (i.e., no sparseness). In addition, to assess the spatial overlap of the decoding maps considering the same retrieval time for all the subjects, we included in the aforementioned test the seven group maps obtained by aggregating the decoding maps at a fixed time point (e.g., group map at the 2 s time point).

#### Assessing the differences between negative and positive memories

The group probability map was obtained by combining the subjects from the two groups, considering the discrimination between “true” and “false” responses irrespectively of the positive or negative emotional valence associated to the retrieved memory. Here we tested whether the different valence of the memories could affect *when* (i.e., the time point with the highest accuracy) or *where* (i.e., the brain regions involved in the process) the retrieval occurred. First, we compared the time points with the highest accuracy between the two groups (Mann-Whitney U test, two-tailed, *p* < 0.05). Second, we measured the spatial overlap within the two groups. To this aim, we first evaluated the spatial overlap of the decoding maps between the 14 subjects using the Sørensen-Dice (SD) coefficient (Dice, 1945; Kolasinski et al., 2016). Subsequently, the Ratio (R) between the averaged SD values within- and the averaged SD values between-groups was computed. R represents whether each group shows a higher within-group similarity (*R* > 1), a higher between-group similarity (*R* < 1), or a spatial overlap between groups (R≅1). Confidence intervals of R were obtained through a permutation test (10,000 iterations, *p* < 0.05).

The multivariate pattern analyses were carried out using Matlab (Matworks Inc., Natick, MA, United States), while Connectome Workbench (Marcus et al., 2011) was used to render the brain meshes in Figure 3.

## Results

### Behavioral results

Response times showed no significant effect for *response* [mean in s ± standard deviation; “True” trials: 1.15 ± 0.22; “False” trials: 1.19 ± 0.20; r *F*_(1, 11)_ = 0.12, *p* = 0.733] or *group* [weddings: 1.21 ± 0.22; funerals: 1.09 ± 0.17; *F*_(1, 11)_ = 1.06, *p* = 0.325], nor for their interaction [*F*_(1, 11)_ = 0.57, *p* = 0.466]. Overall, this evidence indicated that at the button press (i.e., 17.5 s after sentence onset), the retrieval of the autobiographical information was already concluded regardless of the item truthfulness or valence. Response accuracy was at ceiling level (overall accuracy value across conditions: 98%).

### Single-subject decoding results

Since the time required for the retrieval of autobiographical memory may vary among subjects (Svoboda et al., 2006), we avoided a standard group level analysis, focusing only on the single subject decoding of “true” and “false” responses within a relative large time window, from 2 s after trial onset up to 14 s (see Methods). As reported in detail in Table 1 and Figure 2, the decoding was successful in 12 out of 14 subjects (*p* < 0.05), ranging from 65.7 to 86.8%, although it occurred at different time points (mean in s ± standard deviation: 8 ± 4). Averaging the highest accuracies across time points and across all 14 subjects led to an overall mean accuracy of 71.4% with a standard error of 2.0%.

### Group level map

To highlight brain regions involved in the discrimination of “true” and “false” responses, a posterior probability map was built across the whole time window, by combining the single subject decoding maps at the time point with the highest accuracy. The regions involved in the process are depicted in Figure 3 and detailed in Table 2.

**Table 2:** Brain regions, centers of mass (CM) and peak coordinates extracted from the probability map (*p* < 0.33) in Figure 3.

**Regions of the probability map**	**Voxels**	**CM x**	**CM y**	**CM z**	**Peak x**	**Peak y**	**Peak z**
bilateral medial prefrontal cortex	388	0	61	−2	−1	55	−12
bilateral precuneus and posterior cingulate cortex, retrosplenial cortex	365	3	−56	29	6	−48	31
right middle temporal and angular gyri	149	50	−62	23	57	−72	13
bilateral calcarine and right lingual gyrus	124	7	−77	−8	21	−75	−15
left superior frontal gyrus	94	−25	29	52	−22	31	58
right superior and middle frontal gyri	83	25	36	42	30	30	40
left angular gyrus	44	−43	−69	35	−46	−72	34
right supramarginal gyrus	44	62	−42	36	63	−42	34
left superior frontal gyrus	39	−15	48	39	−19	46	37
right anterior insula	26	45	14	−2	45	12	−6
right middle frontal gyrus	26	38	59	2	39	61	4
right middle frontal gyrus	23	41	6	34	41	10	34
right precental gyrus	21	42	−14	50	41	−14	43
left posterior intraparietal sulcus	20	−21	−87	39	−22	−87	40
left middle cingulate cortex and paracentral lobule	20	−4	−32	46	−7	−33	43

By applying a probabilistic threshold of *p* > 0.33 (i.e., the probability of a voxel to be informative in at least 5 out of 14 subjects), irrespectively of timing, a broad set of cortical areas was identified, which comprised several bilateral nodes of the Default Mode Network (DMN), including medial prefrontal, superior frontal and angular regions, retrosplenial cortex, posterior cingulate and precuneus. Precuneus showed the highest overlap among subjects (i.e., nine). In addition, a large cluster was identified bilaterally in early visual cortical areas. Interestingly, in our experiment, other medial temporal lobe key regions, such as the hippocampal and parahippocampal cortex and the amygdala, did not reveal enough discrimination capacity to detect true from false items.

### Reliability of the group level map

Individuals processed the autobiographical memory content with different retrieval times (Svoboda et al., 2006). Therefore, to test whether the cognitive mechanism underlying the discrimination of true and false contents is based on the engagement of the same brain regions across our subjects, we combined single subject decoding maps at different time points showing the lowest sparseness (i.e., highest spatial overlap), to built the best group probability map across subjects. The results, represented in Figure 4, suggest that the best map includes the lowest number of voxels, irrespective of the chosen probability threshold, as compared to a null distribution built by combining different single subject decoding maps at random time points (p < 0.05). Moreover, the seven group maps obtained by aggregating the single subjects decoding maps at each time point fell within the confidence intervals of the null distribution, thus indicating that a standard group level analysis would have led to a non-optimal result.

### Differences between negative and positive memories

First, we examined whether the discrimination between true and false events occurred using brain activity extracted at different time points in the two groups. No temporal differences were found between subjects who retrieved memories from their wedding and subjects who recalled events from the funeral of a loved person. Moreover, we tested whether there was a significant spatial overlap of the decoding maps between the two groups. To this aim, we developed an *ad hoc* measure R, based on the SD coefficient (Dice, 1945; Kolasinski et al., 2016), as detailed in the Methods section (see above). We were not able to demonstrate that the two groups had a specific decoding map, since the R index fell within the confidence interval (*R* = 1.01, 95% confidence intervals: 0.91–1.16).

## Discussion

The present fMRI study was designed to determine whether neural activity can discriminate true from false memories of real autobiographical events, to investigate individual differences in AM processing, and to isolate specific effects of the emotional valence (i.e., positive or negative) on AMs. Given the subjective nature of autobiographical memories, a multivariate technique (Mitchell et al., 2008) was used to evaluate the retrieval process in each subject independently. Results showed that neural activity discriminated AMs in 12 out of 14 participants (mean accuracy ~71%) across a retrieval time of up to 14 s, although discrimination occurred at different time points across subjects. In addition, to overcome single subject differences, we examined the recognition of real AMs also at a group level by combining the individual decoding maps, and highlighted a set of brain regions which mainly overlaps with the AM core network (i.e., medial prefrontal, superior frontal and angular regions, retrosplenial cortex, posterior cingulate, precuneus and early visual areas) described by Cabeza and colleagues (Cabeza and St Jacques, 2007). Finally, we found no specific effects of either positive or negative emotional valence on AMs.

Our experimental approach attempted to investigate individual differences in AM processing using a functional task. Indeed, neuroimaging studies have focused on behavioral scores or trait measures that can account for modulation effects in commonly activated brain areas (Miller and Van Horn, 2007). Usually, these studies included intra-scanner behavioral performance measures, such as accuracy (Callicott et al., 1999; Gray et al., 2003) or reaction time (Rypma et al., 2002; Wager et al., 2005; Schaefer et al., 2006). A small number of studies related brain activation to tasks or measures administered outside of the scanner, including measures of working memory span or fluid intelligence (Gray et al., 2003; Geake and Hansen, 2005; Lee et al., 2006) and measures of personality traits (Gray and Braver, 2002; Kumari et al., 2004). In particular, authors correlated the successful retrieval from episodic (Horn and Miller, 2008; King et al., 2015) or working memory (Rypma and D'Esposito, 2000) with neural activity in specific brain regions. However, only a few studies considered individual variability across the whole brain (McGonigle et al., 2000; Feredoes and Postle, 2007; Seghier et al., 2008).

Several studies showed individual variability in performance and neural activity depending on age (Maillet and Rajah, 2014) and gender (Hill et al., 2014). With respect to AM studies, Piefke and Fink concluded that both factors influence the performance in AM tasks and its underlying neural mechanisms. In particular, aging and gender appear to affect the functional hemispheric lateralization of AM recollection and the degree of involvement of prefrontal, hippocampal, and parahippocampal brain areas (Piefke and Fink, 2005).

As recently demonstrated, individual variability in cognitive strategies during AM retrieval, and particularly the tendency to recollect autobiographical memories from an egocentric perspective, exerted a significant effect on a pivotal region within the AM network, the precuneus, in line with the established role for this region in self-centered representations (Hebscher et al., 2018). Indeed, this recent voxel-based morphometry study showed that larger precuneus volumes were associated with the tendency to recollect autobiographical memories from an egocentric perspective. In addition, Sheldon and colleagues evaluated the impact of individual differences during autobiographical retrieval. Their results showed that self-reported individual differences related to how the subject recalls past events were associated to the intrinsic connectivity between the medial temporal lobe structures and the other nodes of the AM network (Sheldon et al., 2016).

The role of commonalities and differences between subjects, particularly in the time point at which recollection of AMs occurs, needs to be further investigated in order to uncover the association between brain activity and cognitive strategies used to retrieve AMs, as well as with personality traits. Our data showed that the retrieval of AMs relies on the same neural network across subjects, although with individual differences in the time course.

At group level, we evaluated whether neural activity can discriminate true from false autobiographical events, finding a widespread set of brain regions which mainly overlaps with the previously identified AM network (Cabeza and St Jacques, 2007).

The successful recollection from AM is still not fully understood. Rather, several studies investigated the issue of the “feeling of rightness” phenomenon and suggested that the ventromedial PFC could be crucial. Indeed, the activation of this area is commonly observed in tasks requiring self-referential processing (Craik et al., 1999; Gusnard et al., 2001; Kelley et al., 2002) and in decision making tasks under uncertainty, in control processes providing a “feeling of rightness” and in the processing of self-referential information that monitor the veracity of autobiographical memories (Gilboa, 2004).

Other studies have examined the functional networks that subserve the subjective perceptions of familiarity and unfamiliarity in autobiographical recollection. A complex of fronto-parietal regions (lateral PFC and PPC) is involved in cognitive and attentional control processes that guide the recovery of information from memory, as well as in the evaluative processes that monitor retrieval outcomes and guide mnemonic decisions (Tailby et al., 2017).

Interestingly, key medial temporal regions, such as the hippocampal and parahippocampal cortical areas, did not retain enough ability to discriminate between true and false sentences in our experiment. This presumably depends on the adopted task: subjects were presented with sentences that could belong, or not, to the their AM, but differed in one detail only. We speculate that, to monitor the veracity of autobiographical memories, subjects should access their AMs for processing both true and false sentences. Indeed, since the hippocampus is the structure engaged in the initial access to AMs (Daselaar et al., 2008), both types of trial may have recruited it to the same extent.

Since our aim was to investigate which regions of the AM circuit can discriminate true from false AMs, we did not evaluate the recollection of other memories. Thus, we could not exclude that the same neural network could discriminate the truthfulness of other kind of memories.

We also examined whether retrieval of positive and negative emotional events from AM would exert distinctive effects on brain response. First, we assessed whether the discrimination between true and false events in the two groups occurred using brain activity extracted at different time points. No temporal differences were found between subjects who retrieved memories from their wedding and subjects who recalled events from the funeral of a loved one. Moreover, we did not find any significant difference in the spatial overlap of the decoding maps of the two subgroups, thus suggesting that emotional valence did not affected neither the temporal nor the spatial pattern of activity during the retrieval. Indeed, decoding negative and positive autobiographical episodes was a challenging task with fMRI data and in a previous attempt Nawa and colleagues reported accuracies at chance level using an across-participants approach, whereas only half of the sample yielded a significant decoding with a within-participant approach (Nawa and Ando, 2014).

The choice of evaluating the two events (i.e., weddings and funerals) was based on the extensive evidence that emotionally arousing experiences are well-remembered (Brown and Kulik, 2003). Memories of unpleasant occasions, such as an automobile accident, a mugging, or the death of a loved one, are retrieved better than memories of routine days (Pillemer, 1984; Bohannon, 1988; Conway, 1995; Neisser et al., 1996; Sharot et al., 2007). Memories of pleasant occasions, such as birthdays, holidays, and weddings, are also well-retained (Buchanan, 2007). Thus, the strength of the memories of events varies with the emotional significance of the events.

The potential modulatory effect of the valence (either positive or negative) has been previously investigated, but with somehow conflicting results. In some cases, positive events were recalled more easily and directly with respect to negative ones, and led to an increased recovery of peripheral sensory and contextual details (Berntsen, 2002; Schaefer and Philippot, 2005; Kensinger and Schacter, 2006; Ford et al., 2009). The advantage for positive memories seems to be particularly evident when information is self-relevant (Holland and Kensinger, 2010) and some researchers have ascribed it to an overall bias toward accessing positive life experiences (Walker et al., 2003; Berntsen et al., 2011). On the other hand, some studies suggested that positive autobiographical memories are remembered less specifically than negative events (Walker et al., 2003), and that “tunnel memories”—enhanced memory for the central details of an event—are limited to emotionally negative memories. Finally, negative past experiences are remembered with greater emotional intensity than positive memories (Berntsen, 2002).

Our data suggest that monitoring the veracity of highly emotional autobiographical memories requires a unique network of brain regions, irrespectively of the positive or negative valence of the event. In line with previous neuropsychological and neuroimaging evidence, we found that this memory system is mostly right-lateralized. This could reflect the emotional re-experiencing occurring during retrieval and is consistent with findings across different domains that suggest preferential right-hemisphere involvement in emotional and in social cognitive processes (see Svoboda et al., 2006 for a review).

In conclusion, we demonstrated that the entire AM network, with the exception of the medial temporal lobe regions, is engaged in monitoring the veracity of autobiographical memories. This process is mainly influenced by individual differences, rather than by the emotional valence of the experience. In line with previous neuroimaging studies (Miller and Van Horn, 2007), our data confirm that the patterns of brain activity during retrieval of AMs are consistent across subjects, though at different time points. This may be related to the unique manner in which subjects re-experience an autobiographical memory and to the different cognitive strategies used to process information. For this reason, a better understanding of the relationship between AM retrieval and the neural system that underlies this process should rely on the conjoint use of single-subject and group-level data analyses.

## Author contributions

FB, MM, and ER designed the study. FB, MM, DB, and MZ carried out the experiments. FB, DB, GH, AL, PaP, and LC analyzed the data. FB, GH, ER, FL, PiP, and PN wrote the paper, which was approved by all authors. GS, PiP, and PN supervised the project.

### Conflict of interest statement

The authors declare that the research was conducted in the absence of any commercial or financial relationships that could be construed as a potential conflict of interest.
